# Adjustment of Eculizumab Dosage Pattern in Patients with Atypical Hemolytic Uremic Syndrome with Suboptimal Response to Standard Treatment Pattern

**DOI:** 10.1155/2016/7471082

**Published:** 2016-11-29

**Authors:** Camino García Monteavaro, Carmen Peralta Roselló, Borja Quiroga, José María Baltar Martín, Lorena Castillo Eraso, Fernando de Álvaro Moreno, Alberto Martínez Vea, María Teresa Visus-Fernández de Manzanos

**Affiliations:** ^1^Nephrology Service, Hospital San Agustín, Camino de Heros 6, 33401 Avilés, Spain; ^2^Nephrology Service, Hospital Universitari Joan XXIII, C/Dr. Mallafrè Guasch 4, 43005 Tarragona, Spain; ^3^Nephrology Service, Hospital Universitario de Guadalajara, C/Donante de Sangre s/n, 19002 Guadalajara, Spain; ^4^Nephrology Service, Hospitales Madrid, Pl. del Conde del Valle de Súchil 16, 28015 Madrid, Spain

## Abstract

In patients with atypical hemolytic uremic syndrome (aHUS), complement blocking by eculizumab rapidly halts the process of thrombotic microangiopathy and it is associated with clear long-term hematologic and renal improvements. Eculizumab treatment consists of a 4-week initial phase with weekly IV administration of 900 mg doses, followed by a maintenance phase with a 1,200 mg dose in the fifth week and every 14 ± 2 days thereafter. We present three patients with aHUS and suboptimal response to eculizumab treatment at the usual administration dosage who showed hematologic and renal improvements after an adjustment in the eculizumab treatment protocol.

## 1. Introduction

Atypical hemolytic uremic syndrome (aHUS) is a rare chronic disease clinically defined by nonimmune microangiopathic hemolytic anemia, thrombocytopenia, and acute kidney failure [1]. The underlying histological lesion is a systemic thrombotic microangiopathy (TMA) [1] affecting mainly kidney vessels.

aHUS is caused by deregulation of the activation of the alternative pathway of the complement system on cell surfaces. Such deregulation is determined by genetic disorders (mutations and polymorphisms), which decrease the activity of certain complement inhibiting proteins or increase the function of activating proteins [2–7]. Mutations in one or more complement-regulating proteins have been detected in about 60% of aHUS patients [8]. In addition, 6–10% of patients show antibodies targeting the complement factor H (CFH), resulting in decreased CFH activity [7].

For many years intensive treatment with plasmapheresis or plasma infusion was the first-line treatment for aHUS, but still about 33–40% of patients died or required dialysis during the first episode, and one year later 65% showed permanent renal damage requiring dialysis or leading to death [9, 10].

Eculizumab (Soliris®; Alexion Pharmaceuticals, Connecticut, USA) is a humanized monoclonal antibody that binds C5, blocking its split up into C5a and C5b and thus inhibiting the generation of the membrane-damaging C5b-9 complement complex or terminal complement complex (TCC) [11]. In prospective studies the use of eculizumab has been associated with quick and sustained interruption of the complement-mediated TMA and with significant long-term improvements in renal function [12–14]. Currently eculizumab is the new standard of care for aHUS [8, 15].

Eculizumab treatment in aHUS has an initial and a maintenance phase. In the 4-week initial phase, eculizumab IV 900 mg is administered weekly while during the maintenance phase 1,200 mg IV is administered in the 5th week and from that point onwards 1,200 mg every 14 ± 2 days [16].

We present three clinical cases with suboptimal response to eculizumab treatment, which improved after adjusting the dose amount and/or interval.

## 2. Cases Presentation

### 2.1. Case 1

22-year-old female patient was referred to our center due to renal failure, anemia, and thrombocytopenia with the following blood and urine analysis data: blood: creatinine (Cr) 15.3 mg/dL, urea 314 mg/dL, hemoglobin (Hb) 7.3 g/dL, platelets 36,000/*μ*L, lactate dehydrogenase (LDH) 2,975 IU/L, C3 114 mg/dL, and C4 24.5 mg/dL; urine: protein 600 mg/dL and sediment with 10–30 erythrocytes and 2–4 leukocytes/field.

TMA was suspected and aHUS was diagnosed based on the following observations and required tests results: uremia, thrombocytopenia, and hemolytic anemia, Coombs negative with schistocytes; normal immunologic study; negative viral serologies; ADAMTS-13 94%, and negative PCR for Shiga-toxin-producing* E. coli* in feces. At first, no disorders were observed in the genetic analysis and no anti-CFH antibodies were detected. However, an extended test performed some months later revealed a* CFH/CFHR1* hybrid gene (the exon 6 of the* CFHR1* gene is substituted by the exon 23 of the* CFH* gene). Additionally, the patient carries a risk haplotype for aHUS in* CFH* (H3) in heterozygosis and a risk haplotype in* MCP *(MCPggaac) in homozygosis.

Renal biopsy showed signs of acute TMA. Glomerular capillaries and vessels were enlarged and congestive with thrombotic phenomena and erythrocyte fragmentation in the lumen. No signs of vasculitis or sclerosis were observed. Immunofluorescence showed presence of fibrinogen on the wall of some vessels and glomeruli. Light deposits of C1q, C3, and IgM and light chains of Kappa and lambda were found with the same distribution pattern.

Hemodialysis treatment was initiated (6 weekly sessions during the first three weeks, interdaily sessions in the following three weeks, and on-demand hypervolemia from the 6th week onwards) along with plasmapheresis (9 sessions on consecutive days) until the diagnosis of aHUS, at which point plasmapheresis was suspended to start eculizumab treatment (day 10 of hospital admission) at usual doses (Table 1).

Renal function was progressively improved to the point of being able to suspend hemodialysis at 10 weeks of treatment but the improvement in hematologic parameters was delayed. During the initial weeks of treatment LDH remained increased and haptoglobin remained undetectable. At treatment initiation the platelet count was 68,000/*μ*L. After the second dose of eculizumab, platelets reached 214,000/*μ*L but decreased progressively thereafter, and prior to the 6th dose of eculizumab (second maintenance dose: 1,200 mg) the platelet count was 52,000/*μ*L. Thus, the 7th dose of eculizumab was increased to 1,500 mg and it was administered two days earlier. From this point on platelets increased progressively and the following doses were administered according to the usual dose and pattern. After the 8th dose platelets reached 283,000/*μ*L and stayed at levels close to ~200,000/*μ*L from there on.

Five days before the change in the 7th dose of eculizumab, the levels of C3 (103 mg/dL) and C4 (16.5 mg/dL) were within normal ranges (90–180 mg/dL and 10–40 mg/dL, respectively) and the result of the 50% hemolytic complement (CH50) assay was 1.1 mg/dL (normal range: 41–113 mg/dL). At the first determination after increasing the dose of eculizumab the C3 and C4 levels were 104 mg/dL and 30.7 mg/dL, respectively, and the CH50 was 2.2 mg/dL. During the rest of the follow-up, the levels of C3 and C4 remained within normal ranges and CH50 ranged between 0.5 and 1.4 mg/dL.

Ten months after the diagnosis the patient showed a glomerular filtration rate (GFR) of 57 mL/min/1.73 m^2^ and completely normal hematologic parameters.

As a complication, she presented with difficult-to-control arterial hypertension with an episode of hypertensive encephalopathy. However, currently she shows acceptable hypertension control on amlodipine, doxazosin, and furosemide treatment.

### 2.2. Case 2

29-year-old male visited the emergency room (ER) due to overall discomfort. Analyses showed thrombocytopenia, microangiopathic hemolytic anemia (with negative direct Coombs test), and acute decrease of renal function (Cr 31 mg/dL), requiring immediate hemodialysis and a first plasmapheresis session 12 hours after admission. ADAMTS-13 activity was >5%. He was diagnosed with aHUS, showing low levels of C3 (53 mg/dL) and CFH (10.8 mg/dL) and grade 3 malignant hypertension.

Anti-CFH antibodies were not detected in the patient's plasma. The genetic analysis showed two complement-related mutations in heterozygosis: one in exon 2 of the* C3* gene (c193A>C; p.Lys65Gln) and another one in exon 18 of the* CFH *gene (c.2655del; p.Arg885Serfs^*∗*^13).

After two sessions of plasmapheresis, eculizumab treatment was initiated at the third day after admission (Table 2). Response was suboptimal and after 5 weeks of treatment the platelet count had decreased from 110,000/*μ*L to 65,000/*μ*L. Additionally, hemolysis data persisted and the patient presented difficult-to-control hypertension. A renal biopsy was performed 6 weeks after diagnosis, showing data compatible with TMA with 25% chronicity. Immunofluorescence was negative for C1q, C3, IgA, IgG, IgM, Kappa, and lambda deposits.

Given the patient's evolution, the 6th dose of eculizumab was administered 12 days after the 5th dose instead of 15 days afterwards, which is the center's usual protocol according to the Summary of Product Characteristics (SmPC) [16]. After that, analytical data showed improved LDH values, platelet counts, and hemoglobin levels (Table 2). Therefore, usual maintenance dose intervals (15 days) were resumed. Anemia was controlled with iron and erythropoietin, which was suspended once the Hb value was normalized.

During the following weeks serum Cr ranged approximately between 6 and 4 mg/dL but seemed to be in progressive decline. In fact, hemodialysis (which the patient had been given three times a week) could be suspended at the 8th week of treatment with eculizumab (one week after the administration of the 6th dose).

The patient had two later hospital admissions, one due to Hickman catheter-related* S. aureus* bacteremia and another one due to possible respiratory infection plus hypertensive crisis. No reactivation of the aHUS was observed during these infections, although administration of the 8th dose of eculizumab had to be delayed by 6 days because of the first event (no relevant changes in the aHUS clinical and analytical parameters were observed).

Six months after admission the patient presented the following analytical parameters: platelets 149,000/*μ*L, LDH 96 IU/L, Hb 10,7 g/dL, haptoglobin 56 mg/dL, serum Cr 2.37 mg/dL, and GFR 22 mL/min/1.73 m^2^. Mean blood pressure was 147/108 mmHg. Currently arterial pressure is better controlled (~120/85 mmHg) under treatment with five antihypertensive drugs: amlodipine/olmesartan 20 mg/5 mg, doxazosin 8 mg, atenolol 50 mg, and torasemide 5 mg.

### 2.3. Case 3

66-year-old female patient with TMA clinical signs was admitted into our center's ER, transferred from a hospital in the United States. Five days prior to hospital admission in the US, where she was on vacation, she started with progressive asthenia, oral intolerance, and minimum effort dyspnea plus severe headache. According to the US clinical history, on admission she presented with hemolytic anemia (Hb 6 g/dL, LDH > 1,200 IU/L, haptoglobin undetectable, and schistocytes positive), thrombocytopenia (91,000 platelets/*µ*L), and acute renal failure (serum Cr 7 mg/dL). Anti-nuclear antibodies, anti-DNA antibodies, anti-neutrophil cytoplasmic antibodies, anti-glomerular basal membrane antibodies, and rheumatoid factor were all negative. The patient was treated with hemodialysis and plasmapheresis. In addition, she received erythrocyte transfusions (5 concentrates), IV corticosteroids, and 5 daily plasmapheresis sessions before returning to Spain.

On admission to our center, she presented with oliguria, arterial pressure 150/90 mmHg, edema in inferior limbs, ascites, and disseminated crepitation at pulmonary auscultation. Chest X-ray showed bilateral pleural effusions. In laboratory data she showed decreased renal function (serum Cr 4.1 mg/dL), nonimmune hemolytic anemia (Hb 11.0 g/dL; LDH 1,119 IU/L, 7-8 schistocytes per field and direct Coombs test negative) and thrombocytopenia (148,000/*µ*L). C3 and C4 levels were 60.6 mg/dL and 6.7 mg/dL, respectively. The patient started hemodialysis and plasmapheresis while waiting for the results of ADAMTS-13 activity and a renal biopsy for diagnosis. ADAMTS-13 activity was 43%, suggesting the diagnosis of aHUS. The histopathological data from the renal biopsy was consistent with TMA: out of the 20 glomeruli obtained in the biopsy, one was sclerotic and the others showed diffuse lesion with extensive mesangiolysis, sclerosis of capillary loops, and occasional fibrin thrombi; frequent luminal occlusion of helium arterioles with moderate interstitial fibrosis was also observed; and muscular arteries presented with myxoid thickening of the intima with luminal obliteration and presence of fragmented erythrocytes in the parietal zone. Anti-FH antibodies were negative and the genetic complement study showed no disorders.

Eculizumab treatment was initiated according to the SmPC (900 mg/weekly during 4 weeks followed by 1,200 mg every 15 days) along with antimeningococcus prophylaxis (Table 3). Few days after the first eculizumab infusion, the platelet count and the LDH levels worsened and hemodialysis-dependent acute renal failure persisted. Thus, one plasmapheresis session was administered previous to the second infusion of eculizumab. At the third week of treatment, the patient showed dyspnea and X-ray data compatible with acute lung edema with hemodynamic instability to ultrafiltration; as a result she was admitted into the intensive care unit (ICU), where she was intubated and started on continuous venovenous hemofiltration and empiric wide-spectrum antibiotherapy. The patient showed good evolution and negative cultures, except for CMV PCR which was positive, and was treated with ganciclovir, and she was discharged from the ICU one week later.

At the 4th week of treatment, the patient received the last initiation dose of eculizumab (900 mg). However, the platelet count remained low and the levels of LDH remained elevated. Thus, rituximab was initiated at the fifth week, associated with the corresponding first maintenance eculizumab dose (fifth dose), which was increased from 1,200 mg to 1,500 mg. From that point on platelet count improved and LDH decreased (Table 3). Consequently, after the 6th administration of eculizumab the maintenance dosing pattern was back to the usual 1,200 mg every 15 days and the patient was discharged from the hospital 10 weeks after admission. She has remained on hemodialysis up until now, with no new hemolysis data.

## 3. Discussion

Due to genetic and immune reasons, deregulation and overactivation of the alternative complement pathway on cell surfaces underlay the pathogenesis of aHUS. Approximately 60% of patients with aHUS show isolated or combined mutations in genes encoding complement regulatory factors, and 10% present acquired antibodies targeting CFH [1, 17–19]. Mutations include loss-of-function mutations of genes coding for CFH, membrane cofactor protein (MCP), complement factor I (CFI), and thrombomodulin (THBD) and gain-of-function mutations of genes coding for complement factor B and complement component 3 [1, 3, 17, 19–21]. In addition, mutations and genetic polymorphisms of CFH-related proteins (CFHR) may also be the underlying cause of aHUS [21–23]. As a consequence of these alterations, when the complement is activated by a variety of triggering factors (such as infections), its activity on own cells cannot be controlled properly and endothelial damage, inflammation, and thrombosis are caused.

Patient 1 showed a* CFH/CFHR1* hybrid gene and two risk haplotypes for aHUS and patient 2 showed mutations in* CFH* and* C3*, supporting the idea that multiple hits in complement components are necessary for the development of aHUS in some patients [24]. Genomic rearrangements of the* CFH* and the* CFHR* gene region lead to novel fusion proteins and have been previously observed in patients with aHUS [25–28]. The C3 mutation found in patient 2 has been observed to decrease C3b binding to CFH, a fact that may lead to impaired C3b inactivation [29]. However, the* CFH* mutation carried by this patient is not among those commonly reported [30]. The structural and functional consequences of the amino acid change in the protein sequence originated by this mutation are still to be assessed. In contrast, patient 3 did not show any mutations in the complement fraction or anti-CFH antibodies. In this regard, it has been suggested that in this kind of patients aHUS would probably develop due to the existence of still unrecognized genetic or acquired complement disorders [31]. An* in vitro* study showed that sera from patients with aHUS without identified mutations or anti-CFH antibodies still induced more intense C3 and C5b-9 deposits on adenosine 5-diphosphate- (ADP-) activated human microvascular endothelial cells than control sera, which supported the existence of other inherited or acquired complement disorders [31].

The efficacy and safety of eculizumab were firstly established in two 26-week prospective, multicenter, controlled, and open studies, with a mean extension period of 62 weeks, in 37 aHUS patients resistant to plasma exchange/infusion or with chronic plasma exchange/infusion treatment [12]. Clinical benefits regarding hematologic values, TMA-event-free status, and renal parameters were significant at 6 months and were sustained at two and three years of treatment, independently of the mutation status of the patients [13, 14]. The dosing schedule of eculizumab for patients with aHUS was confirmed in those two original trials and proved to be sufficient to achieve and maintain the minimum serum concentration of eculizumab required to block terminal complement activation [12].

Initially our patients did not respond to eculizumab as expected. Although renal function improved progressively in patient 1, platelet counts decreased during the maintenance phase; thus, the 7th dose of treatment was increased (from 1,200 to 1,500 mg) and administered two days earlier in a successful attempt to improve hematologic data. Since the maintenance administration pattern is 14 ± 2 days, although the amount of dose was higher than recommended, the period of administration was still within the SmPC indications [16]. Likewise, patient 2 received the 6th dose three days earlier than the usual center protocol, and since that protocol had a 15-day interval between maintenance doses, the administration at 12 days was also within the SmPC indications. In patient 3, clinical response was poor after the administration of the initiation phase of eculizumab; thus, the first and second maintenance doses were increased from 1,200 to 1,500 mg, and concomitant rituximab was started, showing thereafter an improvement in hematologic data.

Before the marketing of eculizumab, the anti-DC20 chimeric monoclonal antibody rituximab was sometimes used as part of the conventional therapy for aHUS, including support treatment with plasmapheresis in patients with aHUS and anti-CFH antibodies, used in general with low success rates [9]. However, rituximab has sometimes been used successfully, helping platelet recovery and clinical improvement in atypical as well as gemcitabine-related HUS cases [32, 33]. Thus, a possible effect of the administration of rituximab on the improvement observed in patient 3, together with the increases in the fifth and sixth doses of eculizumab, cannot be excluded.

Suboptimal response to eculizumab therapy has been reported in few previous cases. In some Asian patients with paroxysmal nocturnal hemoglobinuria (PNH), a missense mutation in the C5 gene has been associated with poor treatment response [34, 35]. Recently the case of a patient with aHUS carrying a novel truncating CFH mutation in combination with other changes known to be associated with aHUS, who presented frequent relapses of the disease despite eculizumab treatment, has been reported [36]. Therefore, as mentioned, there might still be unrecognized genetic or acquired complement disorders leading to aHUS [31], some of which might respond in a lesser degree to eculizumab treatment.

The complement blockade with eculizumab in 22 patients with PNH has been assessed in a recent study, suggesting that there might be an important variability in the response [37]. The residual functional activity of C5 was screened using a CH50 assay, which is sensitive to changes in the activity of the components of the terminal complement pathway. Lack of detectable CH50 activity (≤10% of normal values) was found in 51% of the samples and was directly related to circulating free eculizumab levels. In this regard, low free eculizumab levels (<50 mg/mL) correlated with detectable CH50 activity, elevated bilirubin levels, and the need for transfusions. The authors have concluded that concomitant assessment of CH50 and circulating free eculizumab levels may be useful for monitoring complement blockade in patients treated with eculizumab in order to detect possible underdosing [37]. Similarly, the determination of the functional alternative complement pathway using a APH50 assay has also been used in the assessment of the response to eculizumab in patients with aHUS [38]. However, it must be taken into account that CH50, which measures the functional activity of the classical complement pathway, is a nonspecific indirect way of measuring the activity of eculizumab and its usefulness in the clinical setting remains controversial [39]. Currently, not all the Spanish centers have included in their aHUS protocols the determination of CH50 or APH50, and the clinical evolution of the patient continues to be a key factor for the assessment of the response to eculizumab and the modification of the treatment pattern.

In this regard, no data is available regarding these parameters for patients 2 and 3. In patient 1, lab analyses showed that CH50 was low after the first six doses of eculizumab (~1 mg/dL), suggesting an effective inhibition of the terminal complement sequence. However, the platelet count continued to decrease and it was decided to increase the 7th dose of eculizumab (which was also administered earlier). As expected, CH50 continued to be low after this treatment change, but an improvement in hematologic data was observed. The levels of C3 and C4 were within normal ranges all the time.

In conclusion, the reason why the patients with aHUS reported in this paper had a suboptimal response to eculizumab remains unknown. However, at least in these cases adjusting the interval of eculizumab administration or increasing the dose were successful strategies. Thus, such temporary management strategies should be considered in patients with aHUS with persistent suboptimal response to eculizumab.

## Figures and Tables

**Table 1:** Eculizumab doses administered to patient 1 and analytical data after the corresponding eculizumab administration.

Phase	Week: dose number	ECU IV (mg)	Platelets (number/*μ*L)	LDH (IU/L)	Hemoglobin (mg/dL)	Haptoglobin (mg/dL)	Schistocytes	Serum creatinine (mg/dL)
Initiation	1: 1	900	68,000	1,270	7.5	<7.6	—	5.3
	2: 2	900	144,000	1,119	10.5	—	—	3.1
	3: 3	900	214,000	1,269	9.8	—	—	5.2
	4: 4	900	141,000	1,135	9.2	<7.4	—	4.0

Maintenance	5: 5	1,200	104,000	—	8.5	<7.4	—	2.9
	7: 6	1,200	52,000	1,092	9.6	<7.4	6 in 10 fields	2.8
	**9**: **7**	**1,500**	62,000	1,099	8.0	<7.4	—	2.1
	11: 8	1,200	85,000	1,037	9.3	<7.4	—	2.1
	13: 9	1,200	283,000	534	7.4	80	—	2.1
	15: 10	1,200	209,000	389	11.3	140	0	3.5
	17: 11	1,200	188,000	342	11.4	91.4	—	2.4

The modified eculizumab dosage pattern is highlighted in bold: the 7th dose of eculizumab was increased from 1,200 mg to 1,500 mg. Also, it was administered 12 days after the 6th (2 days earlier than scheduled according to the center's protocol).

ECU: eculizumab; LDH: lactate dehydrogenase.

**Table 2:** Eculizumab doses administered to patient 2 and analytical data after the corresponding eculizumab administration.

Phase	Week: dose number	ECU IV (mg)	Platelets (number/*μ*L)	LDH (IU/L)	Hemoglobin (mg/dL)	Haptoglobin (mg/dL)	Schistocytes (% in blood)	Serum creatinine (mg/dL)
Initiation	1: 1	900	110,000	317	8	<3	3	12.1
	2: 2	900	116,000	229	9.9	—	<1	13.3
	3: 3	900	62,000	186	10.1	—	—	11.2
	4: 4	900	68,000	—	8.9	—	—	—

Maintenance	5: 5	1,200	65,000	299	7.9	<3	0	8.3
	**7**: **6**	1,200	84,000	—	8.3	—	0	4.7
	9: 7	1,200	131,000	150	9.7	<3	0	5.7
	12: 8^*∗*^	1,200	189,000	143	10.6	—	<0,01	3.7
	14: 9	1,200	208,000	—	13.5	13	—	4.4
	16: 10	1,200	113,000	91	14.6	—	—	4.5
	18: 11	1,200	142,000	117	14.4	36	<0,1	4.1

The modified eculizumab dosage pattern is highlighted in bold: the 6th dose of eculizumab was administered 12 days after the 5th (3 days earlier than scheduled according to the center's protocol).

^*∗*^The administration of the 8th dose of eculizumab was delayed 6 days due to bacteriemia.

ECU: eculizumab; LDH: lactate dehydrogenase.

**Table 3:** Eculizumab doses administered to patient 3 and analytical data after the corresponding eculizumab administration.

Phase	Week: dose number	ECU IV (mg)	Platelets (number/*μ*L)	LDH (IU/L)	Hemoglobin (mg/dL)	Haptoglobin (mg/dL)	Schistocytes per field	Serum creatinine (mg/dL)
Initiation	1: 1	900	93,000	728	8.3	Below the limit	5–8	4.3
	2: 2	900	30,000	1,170	10.5	Below the limit	8	5.6
	3: 3	900	81,000	1,957	9	—	—	6.5^*∗∗*^
	4: 4	900	71,000	1,230	7.8	Below the limit	7–10	0.9

Maintenance	**5**: 5^*∗*^	**1,500**	64,000	1,675	10.5	Below the limit	—	2.3
	**7**: **6**	**1,500**	135,000	1,483	7.9	—	0	2.5
	9: 7	1,200	297,000	794	8.2	249	0	4.3
	11: 8	1,200	198,000	811	9	99	0	5.3
	13: 9	1,200	269,000	560	9.2	—	—	5.2

The modified eculizumab dosage pattern is highlighted in bold: the 5th and 6th doses of eculizumab were increased from 1,200 mg to 1,500 mg.

^*∗*^Initiation of rituximab. ^*∗∗*^The patient was admitted into the ICU due to acute lung edema at the third week of treatment with eculizumab. There she was intubated and started on continuous venovenous hemofiltration and empiric wide-spectrum antibiotherapy. The patient showed good evolution and was discharged from the ICU one week later.

ECU: Eculizumab; LDH: Lactate dehydrogenase; ICU: Intensive care unit.
